# Coastal shoreline change assessments at global scales

**DOI:** 10.1038/s41467-024-46608-x

**Published:** 2024-03-15

**Authors:** Jonathan A. Warrick, Daniel Buscombe, Kilian Vos, Karin R. Bryan, Bruno Castelle, J. Andrew G. Cooper, Mitch D. Harley, Derek W. T. Jackson, Bonnie C. Ludka, Gerd Masselink, Margaret L. Palmsten, Amaia Ruiz de Alegria-Arzaburu, Nadia Sénéchal, Christopher R. Sherwood, Andrew D. Short, Erdinc Sogut, Kristen D. Splinter, Wayne J. Stephenson, Jaia Syvitski, Adam P. Young

**Affiliations:** 1 U.S. Geological Survey, Santa Cruz, CA USA; 2 Marda Science, contractor to U.S. Geological Survey, Santa Cruz, CA USA; 3 New South Wales Department of Planning and Environment, Parramatta, NSW Australia; 4 School of Science, University of Waikato, Hamilton, New Zealand; 5 Univ. Bordeaux, CNRS, Bordeaux INP, EPOC, UMR 5805 Pessac, France; 6 School of Geography and Environmental Sciences, Ulster University, Belfast, UK; 7 School of Agricultural, Earth and Environmental Sciences, University of Kwazulu-Natal, Durban, South Africa; 8 Water Research Laboratory, University of New South Wales, Manly Vale, NSW Australia; 9 Department of Environmental Resources Engineering, Cal Poly Humboldt, Arcata, CA USA; 10 Coastal Processes Research Group, University of Plymouth, Plymouth, UK; 11 U.S. Geological Survey, St. Petersburg, FL USA; 12 Institute of Oceanographic Research, Autonomous University of Baja California, Ensenada, México; 13 U.S. Geological Survey, Woods Hole, Woods Hole, MA USA; 14 School of Geosciences, University of Sydney, Camperdown, NSW Australia; 15 Department of Geology and Geophysics, Woods Hole Oceanographic Institution, Woods Hole, MA USA; 16 School of Geography, University of Otago, Dunedin, New Zealand; 17 Institute of Arctic and Alpine Research, University of Colorado, Boulder, CO USA; 18 Scripps Institution of Oceanography, University of California San Diego, La Jolla, CA USA

**Keywords:** Natural hazards, Ocean sciences, Climate sciences

**arising from** R. Almar et al. *Nature Communications* 10.1038/s41467-023-38742-9 (2023)

During the present era of rapid climate change and sea-level rise, coastal change science is needed at global, regional, and local scales. Essential elements of this science, regardless of scale, include that the methods are defendable and that the results are independently verifiable. The recent contribution by Almar et al.^1^ does not achieve either of these measures as shown by: (i) the use of an error-prone proxy for coastal shoreline and (ii) analyses that are circular and explain little of the data variance.

Here we provide summary information for each of these points:

(i) Although there are a number of satellite-derived shoreline techniques that are published with source code and can be applied to detect the drivers of coastal change at the global scale^2–5^, Almar et al.^1^ have used a simple and error-prone waterline method. Among the problems with waterline proxies, it is widely shown that they are highly dependent on tidal stage over seasonal, annual, and interannual scales because of the intersection of a sun-synchronous data source and astronomical tidal cycles^3,6^. For example, tidal stages for the Landsat imagery used and published openly by Vos et al.^2^ have temporal biases at a range of scales for transects across the globe. Thus, the variability in waterline data is commonly dominated by tidal patterns at a wide range of time scales rather than patterns of coastal change^2–7^.
  The poor quality of waterline measurements can be shown with comparisons with standard techniques. Standard techniques for tracking shoreline position from satellites commonly capture 70–90% of the variance of in situ shoreline measurements (e.g., Fig. S1 in Vos et al.^2^). In contrast, the waterline measurements of Almar et al.^1^ captured only 14–37% (mean = 26%) of the variance of shoreline measurements (Fig. S6). The Almar et al.^1^ method also introduced spurious time-dependent patterns, including 20–50 m of unrealistic shoreline seasonality for Narrabeen Beach, Australia (compare thin lines in Fig. S6i) and a failure to capture the largest accretion event on record, which occurred in 2005 at Torrey Pines, California (compare thick lines in Fig. S6g). Thus, the Almar et al.^1^ methods fail at characterizing local-scale changes, and they provide no evidence whether these failures improve over regional or global scales.
  The Almar et al.^1^ technique also includes only one transect every 0.5°, or every 55,000 m on average, which grossly undersamples the world’s shorelines. In contrast, standard applications of satellite-based shoreline mapping at regional and global scales is conducted at ~100 m transect spacing^2–5,8,9^ in recognition that this scale is required to properly sample the great diversity of coastal settings, behaviors, and geomorphic changes^10–12^. Although space limitations prevent a complete review of the effects of spatial sampling and aliasing for shorelines^7^, we note that down-sampling of 100-m transect data from Vos et al.^2^ to 55,000-m intervals results in fundamentally different distributions of the geomorphic change metrics in these data.
  Almar et al.^1^ do allude to problems of their data, which they describe as ‘hydrodynamic variabilities’ that result in an inability to measure the ‘geological’ shoreline (p.6). And yet, Almar et al.^1^ introduce and summarize their study as relevant to ‘coastal morphological change’ (p.2), ‘shoreline change/evolution’ (p.1-2), and coastal ‘erosion’ (p.6). We argue that if the Almar et al.^1^. technique is unable to measure the landform (or ‘geological’) shoreline, a result we agree with, then nothing can be concluded about landform change, evolution, or erosion.
(ii) The waterline measurements of Almar et al.^1^ were shown to have weak positive correlations with independent water-level factors related to sea level, wave energy, and water discharge from rivers, but only with a globally averaged r^2^ of 0.25 (Fig. 1). That is, a primary finding is that the factors that influence coastal water levels are related (albeit weakly) with the inland position of water on the coastal landscape. We argue that this is a trivial, if not circular, finding. The cross-shore position of the waterline on a beach should be a direct function of the water level. And yet, only ~25% of the variance in Almar et al.’s^1^ waterline data could be explained by this simple relationship. Furthermore, the globally averaged correlation (r) of an ENSO-based model was 0.43 (Fig. 3a). Thus, only ~18% of the variance in the ‘shoreline’ data was explained by ENSO. In light of this low correlation, it should be recognized that tidal stages are significantly correlated with ENSO^13,14^, which raises the possibility that a portion of this correlation results from residual tidal effects in the shoreline data, which as noted above commonly dominate sun-synchronous satellite data. In the end, the waterline method captured only ~25% of the variance in actual shorelines, and the regression analyses only captured 18–25% of the variance in the waterline results. Compounding these results by the quadrature-sum method, it is suggested that only ~5% of the variance of actual shorelines would be explained by the ENSO-based regression models, which is contradictory to the primary conclusions of the paper^1^.

In contrast to the methods and results of Almar et al.^1^, there are numerous studies of regional and global-scale shoreline change from satellite data that have included: (i) methods that are consistent with best practices^7^, and (ii) thorough testing, analysis, and application of shoreline results^2–5,8,9,12^. Additionally, that corpus clearly shows how ENSO plays a complex role in some regions (e.g., Pacific basin^2^), while not playing a role in other regions (e.g., Atlantic coast of Europe^12^).

In summary, we suggest that readers should carefully evaluate these matters and Almar et al.’s general conclusion and headline finding that ENSO is a globally important driver of shoreline change^1^. We look forward to more rigorous analyses of the trends and causes of coastal change from data that have reasonable uncertainties and are published openly as demonstrated by others^2–5,8,9,12^. We point toward studies that not only report scientific results, but also provide public-facing data viewers, data repositories, and source codes as good models for getting information to coastal scientists, managers, and citizens^2,3^. These kinds of information and tools are critical to our understanding of coastal systems and the future of coastal communities during the modern era of population growth, coastal urbanization, climate change, and sea-level rise. Coastal managers and citizenry are looking to the scientific community to provide actionable information at both local and regional scales based on rigorously tested and freely available data. Given the importance of this science, future efforts to increase the understanding of coastal systems and carefully reassess the conclusions of Almar et al.^1^ will be needed.
